# Development and validation of a pseudotyped virus neutralization assay for quantification of anti-Lassa virus neutralizing antibodies

**DOI:** 10.3389/fimmu.2026.1783813

**Published:** 2026-06-16

**Authors:** Edward T. Mee, Emma M. Bentley, Gathoni Kamuyu, Noemi Guerrini, Ali Azizi, Giada Mattiuzzo

**Affiliations:** 1 High Consequence Pathogens (R&D), Medicines and Healthcare products Regulatory Agency, South Mimms, United Kingdom; 2 Coalition for Epidemic Preparedness Innovations, London, United Kingdom; 3 VisMederi Srl, Siena, Italy; 4 Department of Life Sciences, University of Siena, Siena, Italy; 5 Coalition for Epidemic Preparedness Innovations, Washington, DC, United States

**Keywords:** harmonization, Lassa virus, neutralization assay, pseudotyped virus, validation

## Abstract

**Background:**

Lassa virus is endemic in many West African countries and has significant epidemic/pandemic potential. The Coalition for Epidemic Preparedness Innovations (CEPI) Centralized Laboratory Network (CLN) is developing validated, harmonized methods to support and accelerate vaccine development in the event of an outbreak. We present here the development and validation of an assay to quantify anti-Lassa virus neutralizing antibodies. As the method uses a pseudotyped virus (PV) instead of authentic Lassa virus—a pathogen listed as hazard group 4—it can be performed at a lower containment level, making it widely accessible especially in those regions where Lassa fever is circulating.

**Methods:**

A recombinant vesicular stomatitis virus (rVSV)-based pseudotyped virus neutralization assay (PVNA) was developed. A working standard was prepared and calibrated against the International Standard (IS) for Lassa virus neutralizing antibodies, enabling results to be expressed in International Units. The method was validated according to the International Council for Harmonisation (ICH) Q2(R2) guidelines.

**Results:**

The rVSV-based PVNA was successfully developed and validated. The analytical range of the assay was established as 10–100 IU/mL, and seropositive samples from a Lassa vaccine clinical trial fell within this range. Results from the PVNA showed direct correlation with results obtained from authentic virus neutralization methods. The method was, therefore, concluded to be fit-for-purpose.

**Conclusion:**

We have successfully developed and validated a PVNA for the quantification of neutralizing antibodies against Lassa virus. We have also provided a practical example of how to calibrate a serological method to the WHO International Standard to achieve greater harmonization and comparability between studies. The method is now being transferred to partner laboratories within the CLN to enable harmonized testing of clinical trial samples and support the development and evaluation of Lassa vaccine candidates.

## Introduction

Lassa virus, the causative agent of Lassa fever, is listed as a priority pathogen by both the World Health Organization (WHO) Research and Development (R&D) Blueprint for Epidemics and the Coalition for Epidemic Preparedness Innovations (CEPI), because of its epidemic potential and lack of a licensed vaccine (1, 2). Several vaccine candidates are currently being evaluated in pre-clinical and phase I studies, and one candidate advanced to a phase II clinical trial in April 2024 (3).

Lassa fever is endemic in several West and Central African countries and can cause severe and sometimes fatal symptoms. Lassa virus is classified as hazard group 4 and therefore must be handled in high-containment facilities, complicating vaccine development efforts, particularly the assessment of the protective potential of vaccine-induced immune responses. Neutralizing antibodies are often used as a surrogate/correlate of protection during vaccine development and evaluation (4). Neutralization of authentic Lassa virus is hampered by the stringent precautions necessary for handling the virus and limited access, affordability, and throughput of such methods. For late-stage clinical trials, thousands of samples would typically be tested, which would be impractical in a Containment Level (CL)-4 environment.

In recent decades, the development of pseudotyped viruses (PVs) has facilitated our ability to assess virus neutralizing responses without the need for handling authentic viruses (5). The envelope protein of the virus of interest is displayed on the surface of an unrelated virus such as human immunodeficiency virus (HIV) or vesicular stomatitis virus (VSV). The genome of the backbone virus, e.g., HIV or VSV, is modified to partially remove viral genes, such as their own envelope protein gene, resulting in a PV with receptor tropism similar to the authentic virus, but which is non-pathogenic and can be handled safely at CL-2 (6). The incorporation of a reporter gene such as luciferase into the PV genome enables automated, quantitative assessment of the viral infection and, conversely, neutralization. PV can, therefore, be used as an alternative to the authentic virus in methods measuring neutralizing activity targeting the virus of interest’s envelope protein; it is important, however, to demonstrate that the pseudotyped virus neutralization assay (PVNA) measures a biologically relevant parameter, i.e., the ability of the sample to neutralize authentic virus. A strong correlation is often seen between neutralizing antibody titers obtained using pseudotyped and authentic viruses (7), though it must be empirically demonstrated for each virus.

CEPI established a Centralized Laboratory Network (CLN), currently comprising 20 partners, to accelerate vaccine candidate evaluation during the COVID-19 pandemic (8–10). Central to the program is the development, validation, and technical transfer of harmonized quantitative methods for assessment of vaccine immunogenicity. This includes methods for quantification of (i) binding antibodies [ELISA, Luminex, and Meso Scale Discovery (MSD)], (ii) neutralizing antibodies [Virus Neutralization Assay (VNA) and PVNA], and (iii) cellular immunity [Enzyme-Linked ImmunoSpot (ELISPOT)]. This approach enables the comparison of clinical trials performed at different geographical sites and times, without the confounding factors of different methodology, assay readouts, and testing lab proficiency. The initial phase of the project enabled the qualification of multiple methods for evaluation of COVID-19 vaccine candidates at seven laboratories (11) and the completion of approximately 130,000 assay runs from over 60 clinical trials (12). The CLN has now expanded its scope to develop methods for several priority pathogens, establishing new methods and a global capacity that can support more rapid vaccine development in the event of an epidemic or pandemic (13, 14).

Methods used in the evaluation of clinical trial samples must be demonstrated to be fit-for-purpose, qualified, or validated, prior to use, according to sample type and the trial stage. An additional, highly desirable, criterion is that the method—or the results generated from it—is standardized and harmonized to enable comparison of studies performed in different laboratories or geographical areas (12). Standardization of methods within the CLN is achieved by sharing critical reagents, such as reference standards and control materials. Harmonization is further supported by the use of common standard operating procedures, reagents, aligned data analysis approaches, and a robust analytical method transfer process. Nevertheless, because of differences between laboratories (e.g., equipment, procedures, different staff, and non-critical materials), some bias in numerical results between laboratories may still occur (11). Reporting the biological potency of the clinical samples using a common, internationally recognized unitage reduces inter-laboratory variation and facilitates the comparison of results with laboratories outside the CLN (15, 16). WHO International Standards are the highest order of reference material and serve as primary calibrants for biological assays, allowing for results to be expressed in International Units [IU (17);]. In 2021, the WHO Expert Committee on Biological Standardization established the First International Standard for anti-Lassa virus antibody and an International Reference Panel following a multi-center collaborative study for the harmonization of methods quantifying antibody responses to Lassa virus infection or vaccination (18).

Antonelli et al. (19) recently described the validation of an HIV-based PVNA for the assessment of anti-Lassa virus neutralizing antibodies. Here, we describe the validation of a VSV-based PVNA, with several key advantages over the previously described work. First, the inclusion of a working standard (WS), calibrated against the International Standard for Lassa virus antibodies, enables the results to be expressed in IU/mL. This will allow data comparison between different studies and methods (20). Second, we confirmed the biological relevance of the method by comparing the neutralizing activity of a set of samples measured by the PVNA method with the results obtained from neutralization methods against authentic Lassa virus. In addition, VSV-based systems can be used in populations with high levels of antiretroviral therapy use, which can lead to overestimation of neutralizing antibodies in retrovirus-based PV systems (21–23).

Our results demonstrated that the VSV-based PVNA is suitable for quantification of anti-Lassa virus neutralizing antibodies and has been adopted by the CEPI CLN. The method is currently being transferred to two CLN laboratories in Africa, the Institut Pasteur of Dakar in Senegal and Synexa Life Sciences in South Africa, to support future clinical trials for Lassa fever vaccines.

## Methods

### Cell lines and plasmids

HEK293T/17 (ATCC CRL-11268) and Vero (ATCC, CCL-81) cells were maintained in Dulbecco’s Modified Eagle Medium (DMEM) containing GlutaMax™ (Invitrogen), supplemented with 10% (v/v) heat-inactivated fetal bovine serum (FBS; Pan Biotech BmgH), 100 U/mL penicillin, 0.1 mg/mL streptomycin, and 10 mM 4-(2-hydroxyethyl)-1-piperazineethanesulfonic acid (HEPES, Merck, UK). The LASV-GP plasmid was kindly provided by Prof. Teresa Lambe, University of Oxford. Briefly, the codon optimized sequence of the LASV Josiah strain (Lineage IV) GP gene (GenBank accession number: NP_694870.1) was synthesized and cloned in the pCAGGS plasmid using EcoRI and XhoI restriction sites.

### Production of LASV–rVSV pseudotyped virus

Production was performed following the method described by Whitt (24). HEK293T/17 cells were seeded at 4 × 10^5^ cells/mL (10 mL total) in 100 × 20 mm cell culture treated dishes. Cells were incubated overnight at 37 ± 1 °C, 5% ± 1% CO_2_, and ≥85% relative humidity and confirmed to have reached 80%–90% confluence after 24 ± 2 h. Cells were transfected with 12 µg of pCAGGS-LASV GP plasmid and 60 µL of 1 mg/mL polyethyleneimine, branched (PEI, Sigma-Aldrich) in 200 µL of Opti-MEM (Invitrogen). After 20-min incubation at ambient temperature, the PEI/DNA mix was added dropwise with gentle rocking to the HEK293T/17 cells. After 24 h, the media was removed and replaced with 5 mL of serum-free DMEM containing a recombinant glycoprotein-deficient VSV with the luciferase gene [G*ΔG-rVSV(Luc), Vector Builder, UK] at a multiplicity of infection (MOI) of 0.1. Cells were incubated at 37 °C for 2 h, media was discarded, and cells were washed five times with 3 mL of phosphate buffered saline (PBS). Fresh media (8 mL) was added, and dishes were incubated for a further 24 h at 37 ± 1 °C, 5% ± 1% CO_2_, and ≥85% humidity. Supernatant of infected cells was harvested and filtered through a 0.45-µm filter. Single-use aliquots of the PV were prepared and immediately frozen at −80 °C.

### Titration of LASV–rVSV pseudotyped virus

Vero cells were seeded in 96-well flat-bottom plates, at 2 × 10^4^ cells per well and incubated at 37 ± 1 °C, 5% ± 1% CO_2_, and ≥85% relative humidity for at least 3 h. LASV recombinant vesicular stomatitis virus (rVSV) PV stocks were first diluted in tubes to 1:50 or 1:250, followed by seven 5-fold serial dilutions in cell growth media as described above. Following dilution, 100 µL of each dilution was inoculated onto the previously seeded Vero cells. Cells were incubated for 24 ± 2 h at 37 ± 1 °C, 5% ± 1% CO_2_, and ≥85% relative humidity. Supernatant was discarded and infectivity was determined based on luciferase activity using BrightGlo luminescent substrate (Promega, UK). The substrate was equilibrated to ambient temperature and mixed at a 1:1 volume with DMEM without phenol red (Gibco, UK). Supernatant from infection plates was removed by aspiration and discarded. The BrightGlo substrate was immediately added (100 µL per well) and incubated at ambient temperature for 5 ± 2 min. Each well was mixed once, and 85 µL was transferred to a white, opaque, 96-well flat-bottom plate. Luminescence was acquired using the GloMax Discover system (Promega, UK) with an integration time of 0.3 s and expressed in relative light units (RLU). All wells exhibiting RLU values greater than 10 times the background (mean of uninfected cells) were scored positive. The virus titer was calculated as the TCID_50_ using the Spearman–Karber method (25).

### Plasma samples

The First WHO International Standard for anti-Lassa fever virus antibodies (NIBSC code 20/202) and WHO International Reference Panel for anti-Lassa fever virus antibodies (NIBSC code 21/332) were produced and distributed from the Medicines and Healthcare products Regulatory Agency on behalf of the WHO (18). These comprise lyophilized plasma from convalescent patients with Lassa fever. Each ampoule was reconstituted in 0.25 mL of sterile ultrapure water according to the product instructions for use. Seven additional seropositive samples used in a feasibility study for the production of a WS were also available. These were aqueous frozen samples from convalescent patients with Lassa fever from Sierra Leone (18). Seronegative plasma from healthy donors with no known exposure to Lassa virus were obtained from the USA and UK. All the samples were collected with informed consent, and ethical approval was given by the relevant bodies as indicated in (18, 26). A full list of samples is provided in **Table 1**. Serum pools from clinical trial volunteers were provided by the International AIDS Vaccine Initiative (3).

**Table 1:** Lassa seropositive and seronegative samples.

Sample ID	Sample type
20/246	WHO Panel 21/332
20/228	WHO Panel 21/332
20/222	WHO Panel 21/332
20/202	WHO International Standard
20/204	WHO Panel 21/332
20/226	WHO Panel 21/332
20/244	WHO Panel 21/332
20/248	WHO Panel 21/332
LASV-2	CEPI Panel
LASV-5	CEPI Panel
LASV-11	CEPI Panel
LASV-13	CEPI Panel
LASV-17	CEPI Panel
LASV-19	CEPI Panel
LASV-7	Negative
LASV_NEG_01	Negative
LASV_NEG_02	Negative
LASV_NEG_03	Negative
LASV_NEG_04	Negative
LASV_NEG_05	Negative
LASV_NEG_06	Negative
LASV_NEG_07	Negative
LASV_NEG_08	Negative
LASV_NEG_09	Negative
LASV_NEG_11	Negative
LASV_NEG_12	Negative

### Preparation and calibration of working standard

WS was produced by combining high-titer convalescent plasma from Lassa fever-recovered individuals from Nigeria and diluting approximately threefold in human plasma unreactive for Lassa virus to achieve a concentration that resulted in a typical neutralization curve that approached both the upper and lower asymptotes. Single-use aliquots were prepared, frozen at −80 °C, and used throughout all subsequent work. The WS was calibrated by preparing serial dilutions of the IS and WS from 1:20 to 1:640 and testing them in parallel in the PVNA. RLU values were normalized and relative potency of the WS was calculated in SoftMaxPro 7.1 (Molecular Devices, UK) using parallel line (global four-parameter logistic fit) analysis.

### Pseudotyped virus neutralization assay

**Figure 1:**
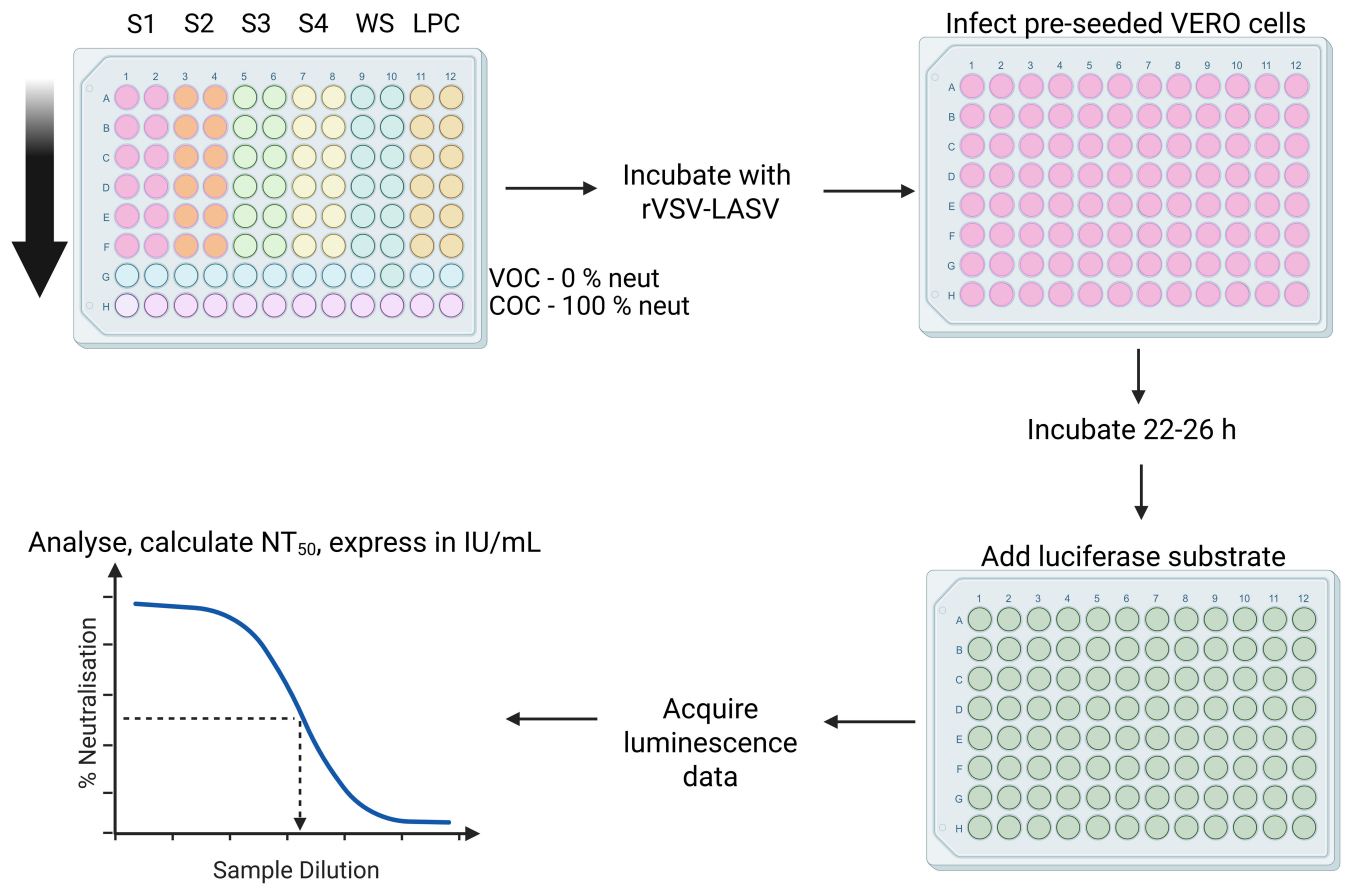
Schematic of PVNA. S1–S4 indicate sample locations; WS, working standard; LPC, low positive control; VOC, virus-only control; COC, cell-only control. Created in BioRender. Mee, E. (2026). https://BioRender.com/56vkp89.

A schematic of the method is shown in **Figure 1**. Vero cells were harvested at 85%–100% confluence, counted using a Countess II system (Invitrogen, UK) and diluted to 5 × 10^5^/mL in complete media and seeded at 100 µL per well into 96-well flat bottom plates. To minimize edge effects, cells were allowed to settle at ambient temperature and atmosphere in a humidified, vented box for 60–90 min before incubation at 37 ± 1 °C, 5% ± 1% CO_2_, and ≥85% humidity for at least 3 h.

LASV–rVSV PV was diluted in DMEM containing Glutamax™, supplemented with 2% (v/v) heat-inactivated FBS, 100 U/mL penicillin, 0.1 mg/mL streptomycin, and 10 mM HEPES to a working concentration of 6,667 TCID_50_/mL (corresponding to 400 TCID_50_/well input volume). Antibody samples were twofold serially diluted in 96-well U-bottom plates from 1:10 to 1:320. A 60-µL volume of virus working dilution was added to each sample well plus to virus-only control (VOC) wells containing only media. The final dilutions of each sample were, therefore, 1:20 to 1:640. An additional 60 µL of media was added to the cell-only control (COC). The WS and a low positive assay run control were included on every plate and processed in parallel with samples. Neutralization plates were then incubated at 37 ± 1 °C, 5% ± 1% CO_2_, and ≥85% humidity for 60 ± 15 min. Media was removed by aspiration and discarded. The incubated antibody/virus mixtures were inoculated (100 µL per well) onto the cells. Plates were then returned to the vented humidified box and incubated at 37 ± 1 °C, 5% ± 1% CO_2_, and ≥85% humidity for 24 ± 2 h. Following incubation, infectivity was measured by the addition of the BrightGlo luminescence substrate as described above. Data were analyzed using a validated template in SoftMaxPro GxP v7.1 (Molecular Devices, UK), based on the method described by Ferrara et al. (27). Arithmetic means of VOC and COC were calculated and used to define 0% (*N*_min_) and 100% (*N*_max_) neutralization, respectively. All sample and standard wells were then normalized using the equation:


$$\%\;neutralization=\frac{RLU-N_{\min}}{N_{\max}-N_{\min}}*100$$

where *RLU* = Relative Light Units in well, *N*_min_ = RLU at minimum (0%) neutralization, and *N*_max_ = RLU at maximum (100%) neutralization.

A four-parameter logistic curve was fitted to each sample or standard using the formula:


$$y=D+\;\frac{A-D}{1+\;{\left(\frac{x}{C}\right)}^{B}}$$

where *x* = sample dilution, *y* = % neutralization, *A* = Lower asymptote, fixed at 0, *B* = Hill Slope, *C* = NT_50_, and *D* = Upper asymptote, fixed at 100.

Results were expressed in IU/mL using the conversion:


$$\text{Result }(\text{IU}/\text{mL})=\frac{{\text{Observed Sample NT}}_{50}}{{\text{Observed Working Standard NT}}_{50}}*\text{Assigned Working Standard }(\text{IU}/\text{mL})$$

### Validation of PVNA

Validation was performed according to the International Council for Harmonisation (ICH) Quality Guideline 2, Revision 2 (28). A summary of performance characteristics and acceptance criteria is provided in **Table 2**.

**Table 2:** Summary of PVNA performance characteristics.

Performance characteristic	Acceptance criteria	Result
Accuracy	Observed results within 50%*–*200% of expected results in the range 20–100 IU/mL	Observed results within 50%*–*200% of expected results in the range 10–100 IU/mL
Dilutional linearity	Regression *R*^2^ ≥ 0.90 and slope = 0.8 to 1.20 across the range 20–100 IU/mL	*R*^2^ = 0.9695, slope = 1.026 across the range 10–100 IU/mL
Specificity	Specificity ≥ 90%	100% (78.2%*–*100%)
Sensitivity	Sensitivity ≥ 90%	100% (75.3%*–*100%)
Selectivity	GCV< 25% for samples mixed with anti-RVFV-positive sample or Ig-depleted serum	15.20%
	Anti-RVFV-positive sample below LOD	<LOD
Repeatability	GCV ≤ 50%	12.12%
Intermediate precision	GCV ≤ 50%	28.28%
Analytical range (LLOQ, ULOQ)	LLOQ ≤ 20 IU/mL ULOQ ≥ 100 IU/mL	10 IU/mL 100 IU/mL
Detection limit	DL ≤ 20 IU/mL	8.60 IU/mL

### Comparison of PVNA with authentic virus neutralization assay

Seven positive samples included in the current study had previously been tested by authentic virus neutralization assays as part of the evaluation of the International Standard for anti-Lassa virus antibodies 20/202 (18). The inclusion of the International Standard alongside the seven samples enabled results to be expressed in IU/mL. Of eight laboratories that tested the samples, six employed assays using the Josiah strain of Lassa virus. One laboratory identified only two of the seven samples as positive; results from this laboratory were, therefore, excluded from further analysis. The geometric mean of the remaining five laboratories was calculated and compared to the results obtained by PVNA by Pearson correlation and Bland–Altman analysis of log_10_-transformed data.

### Preliminary assessment of method robustness

Three positive samples plus the low positive control were tested in parallel with deliberate modifications to conditions. The impact of cell passage (P + 2–P+20), cell seeding density (3–7 × 10^5^/mL), ambient temperature incubation time (60–90 min), neutralization time (45–75 min), and duration of infection (22–26 h) were assessed. Two additional samples were subjected to 5 or 10 rounds of freezing at −80 °C and thawing, then tested alongside a control sample with no additional freeze–thaw cycles.

### Data analysis

Statistical analysis was performed using MiniTab 18. Variability was expressed as geometric coefficient of variation (% GCV) calculated according to the formula $\%\text{GCV}=100\times \left(10^{\sigma }-1\right)$, where σ is the standard deviation of the log_10_-transformed data. Data visualization was performed using GraphPad Prism 10.

## Results

### LASV rVSV PV production, stability, and application in PVNA

VSV-based LASV PV particles were produced by infection of HEK293T cells expressing LASV Josiah lineage glycoprotein with a non-replicative VSV with the gene for the glycoprotein replaced by a marker gene, firefly luciferase. The LASV rVSV PV stock titer on Vero cells was calculated as 1.12 × 10^7^ TCID_50_/mL.

**Figure 2:**
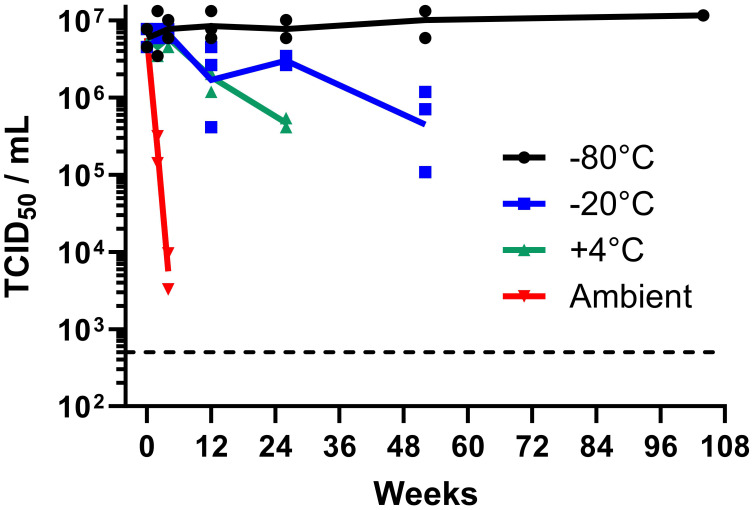
Evaluation of rVSV-LASV stability over a 2-year period. Symbols indicate individual replicates (n = 2–3), and lines indicate geometric mean. The dashed line indicates assay detection limit based on the dilution scheme.

Single-use aliquots were prepared, and stability of the PV was assessed by storage at temperatures from −80 °C to ambient (~+20 °C) for up to 2 years. No loss in titer was observed for the samples stored at −80 °C. A loss of 1–2 log_10_ TCID_50_ was observed after 1 year at −20 °C. More rapid degradation was observed in samples stored at +4 °C or ambient temperature (**Figure 2**).

The LASV–rVSV was used instead of the authentic virus in a neutralization method based on a 96-well plate format in which antibody samples are twofold serially diluted and tested in parallel (**Figure 1**). Up to four samples can be assessed per plate. Each plate includes a WS, which serves as calibrant, and neutralizing activity of the samples is reported relative to that of the WS. Each plate also includes a low-titer positive control (LPC) to assess run validity. The LPC is a high-titer convalescent plasma sample diluted in negative plasma to a concentration close to the limit of detection. The LPC is monitored in every plate and must fall between empirically defined limits based on the results from initial experiments.

### Calibration of working standard against the WHO International Standard for anti-Lassa virus antibodies

**Figure 3:**
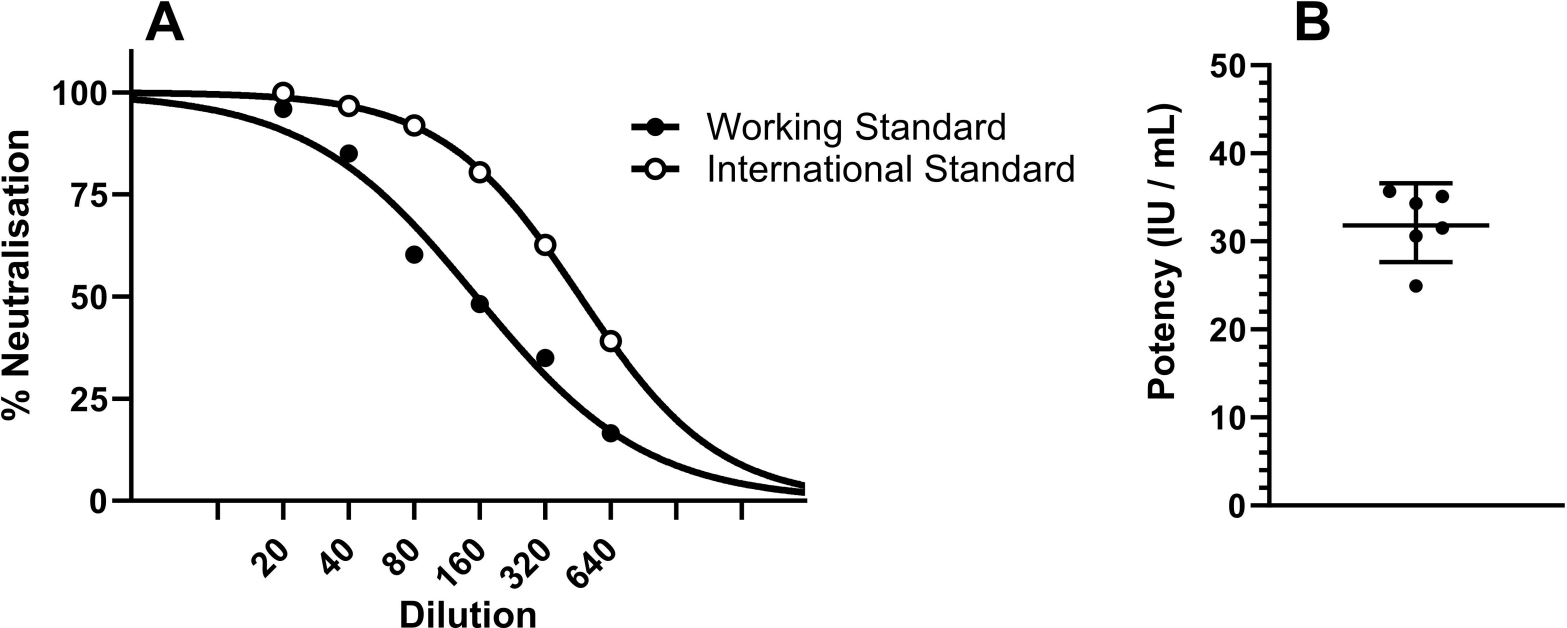
Calibration of working standard. **(A)** Representative curves showing neutralizing response of dilutions of International Standard 20/202 and working standard. Symbols indicate the mean of duplicate samples. Lines indicate fitted four-parameter logistic regression. **(B)** Potency estimates for six independent calibration runs. Lines indicate geometric mean and 95% confidence intervals.

Inclusion in every assay of a WS, calibrated against the WHO International Standard, and reporting results relative to it, is essential to express sample potency in IU, therefore enabling comparison across different runs, laboratories, and methods measuring the same analyte. A WS was prepared from a pool of Lassa fever convalescent plasma and calibrated against the International Standard following WHO guidelines (29). Serial dilutions of the WS and IS 20/202 were run in parallel in the PVNA, and the value of the WS for each experiment was calculated by parallel line analysis. To increase confidence in the WS, six independent runs were performed, and the geometric mean was assigned as the value of the WS. Data are summarized in **Figure 3**. The WS was determined to have a neutralizing titer of 31.8 IU/mL (95% CI 27.6–36.6 IU/mL).

### PVNA accuracy and dilutional linearity

**Figure 4:**
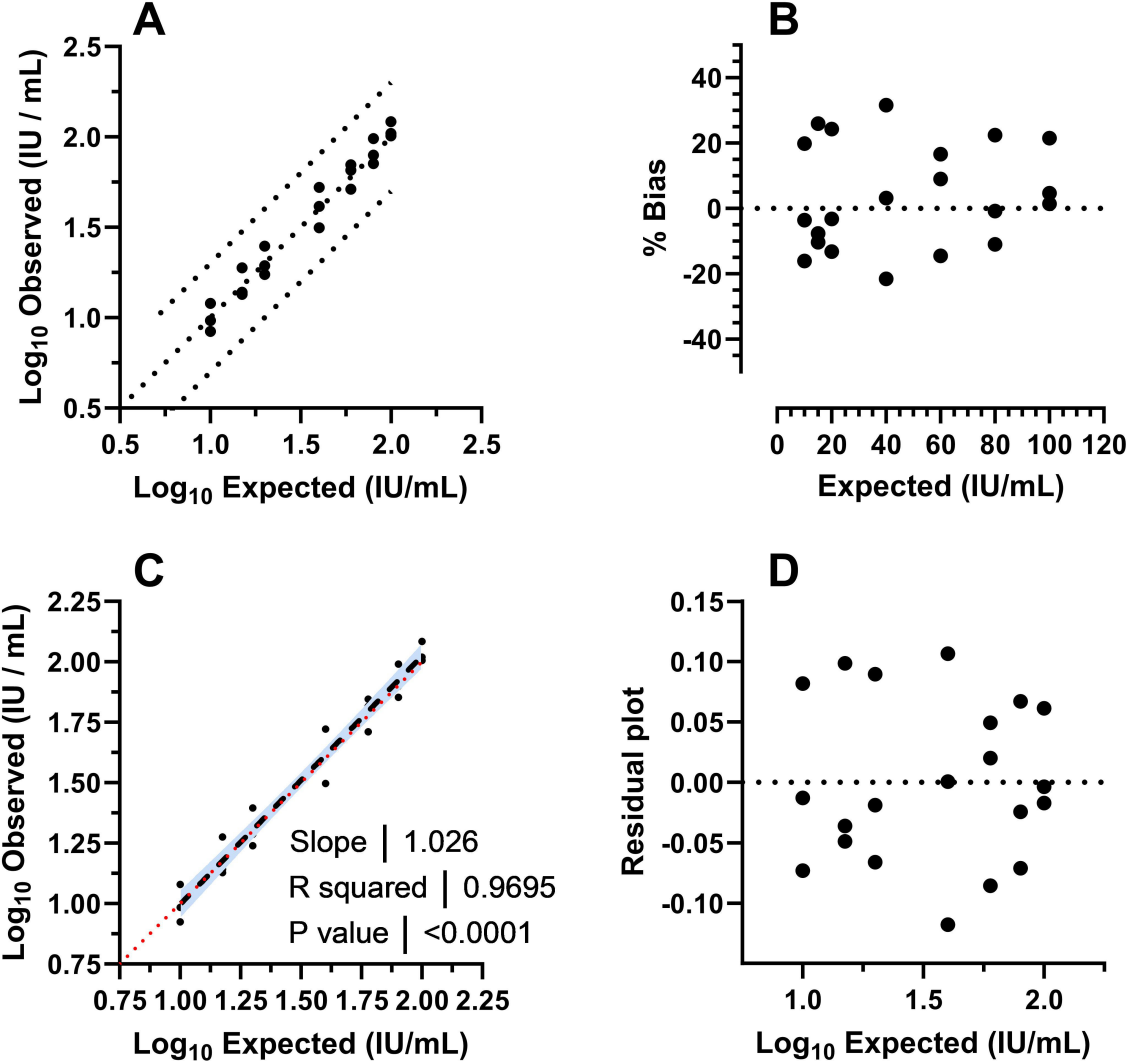
Assessment of method accuracy and linearity. **(A)** Observed versus expected titer for the spike recovery panel. Dotted lines indicate perfect concordance and 50%–200% intervals. **(B)** Difference between the observed and expected values. **(C)** Observed versus expected titer for the spike recovery panel. The dashed line indicates slope, and blue shading indicates 95% confidence band of regression line. The red dotted line indicates equivalence (slope = 1). **(D)** Residuals from the fitted regression line.

The PVNA method was validated following ICH Q2(R2) guidelines (28), and performance characteristics with acceptance criteria are summarized in **Table 2**. Accuracy and dilutional linearity were assessed in parallel using a panel generated by spiking of the International Standard (20/202) into negative human plasma at concentrations from 5 to 100 IU/mL. Samples were then tested in three independent runs. Samples at 5 IU/mL were all undetectable. Samples from 10 to 100 IU/mL were included for further analysis. All spike recovery samples fell within the 50% to 200% acceptance limits, with no trend toward higher or lower bias at the extremes (**Figures 4A, B**). Dilutional linearity was assessed using the same data set. A regression slope of 1.026 was observed (within the acceptance criterion of 0.80–1.20), with *R*^2^ of 0.9695 and evenly distributed residuals (**Figures 4C, D**). The method was, therefore, confirmed to be accurate and linear in the range 10–100 IU/mL.

### PVNA sensitivity, specificity, and selectivity

Sensitivity and specificity were assessed in parallel using a panel of 15 positive and 13 negative samples (**Table 1**). Samples were considered as true positives based on the results of the collaborative study undertaken to support the establishment of the IS and Reference Panel (18), with the majority of independent laboratories reporting a positive result using a pseudotyped or authentic virus neutralization assay. The expected results were observed for all positive and negative samples. The method met the acceptance criteria (≥90%) for both sensitivity and specificity (**Table 2**). Confidence intervals were wider than ideal due to the limited number of available samples. It is expected that confidence intervals would converge at greater than 90% with the availability of additional known positive and negative samples. Selectivity was determined by spiking a Lassa seropositive sample into either Ig-depleted human serum or serum positive for Rift Valley fever virus (RVFV), a virus in the same order *Hareavirales* within the *Bunyaviricetes* class. Similar titers were obtained with both samples, with a GCV of 15.2%, indicating no appreciable interference. The RVFV serum sample alone was non-reactive in the LASV PVNA (**Table 3**).

**Table 3:** Summary of expected and observed results for selectivity assessment.

Panel ID	Titer (IU/mL)
Seropositive 1:2 in Ig −ve serum	46.49
Seropositive L2 1:2 in RVFV seropositive serum	56.82
RVFV seropositive serum	NEG

### PVNA precision

Precision was expressed in terms of repeatability and intermediate precision. Repeatability was assessed by testing three samples of varying titer. Each sample was tested in triplicate within a single plate. Data were log_10_-transformed and analyzed using a mixed-effects analysis of variance (ANOVA) with replicate as a random factor and sample ID as a fixed factor. The overall repeatability was expressed as a GCV of 12.12%, meeting the acceptance criterion of ≤50% (**Table 4**). Intermediate precision was assessed by testing four samples on each of 3 days by each of two operators. Results were log_10_-transformed and analyzed using a mixed-effects ANOVA with operator and day (nested within operator) as random factors and sample as a fixed factor. The ANOVA model indicated a negligible contribution from operator. The major sources of variance were inter-day variance and error (biological variation). Overall intermediate precision was expressed as a GCV of 28.28% (**Table 5**), meeting the acceptance criterion of ≤50%. .

**Table 4:** Summary of variance components for repeatability estimation.

Source	Var	% of Total	p-value	%GCV	Validation target	Result
Error	0.002468	100%	0.042	12.12%	GCV ≤ 50%	Pass

**Table 5:** Summary of variance components for intermediate precision estimation.

Source	Var	% of total	p-value	%GCV	Target	Result
Operator	0	0.00	–	–	N/A	N/A
Day (operator)	0.005627	48.11%	0.108	18.85%	N/A	N/A
Error	0.006069	51.89%	0.003	19.65%	N/A	N/A
Total	0.011695	–	–	28.28%	GCV ≤ 50%	Pass

### PVNA analytical range and limit of detection

The analytical range of a method is defined by the highest and lowest samples that satisfy the accuracy, linearity, and intermediate precision criteria, also known as upper and lower limit of quantification (ULOQ and LLOQ), respectively. The accuracy and dilutional linearity data were re-assessed for precision by determining the GCV of the three independent determinations (**Table 6**). The sample at 5 IU/mL was not detectable. The GCV of all other spike recovery dilutions was ≤30%, within the precision acceptance criterion of ≤50%. Hence, the analytical range of the assay was determined to be 10–100 IU/mL. Another important parameter that is distinct from the LLOQ is the limit of detection (LOD), defined as the lowest concentration sample generating a positive signal. The signal was considered positive if it met three criteria: an NT_50_ of >20 (based on an initial sample dilution of 1:20), *R*^2^ ≥ 0.750, and a minimum of one sample dilution resulting in >50% neutralization. Based on the spike recovery data, a sample with an expected titer of 5 IU/mL did not generate a valid signal. The lowest true-positive sample generated a titer of 8.60 IU/mL, satisfying the criterion of a LOD ≤ 20 IU/mL. Together with the analytical range results, this indicates that the method retains a high level of accuracy and precision even for samples close to the LOD.

**Table 6:** Summary of results used for the determination of analytical range.

Sample	Expected titer (IU/mL)	Observed titer^a^ (IU/mL)	% of expected (target 50%–200%)	GCV of replicates (target ≤ 50%)	Result
SR_01	100	108.85	108.85%	10.15%	Pass
SR_02	80	82.13	102.66%	17.49%	Pass
SR_03	60	61.70	102.83%	17.76%	Pass
SR_04	40	40.86	102.15%	29.42%	Pass
SR_05	20	20.30	101.50%	20.23%	Pass
SR_06	15	15.21	101.40%	20.78%	Pass
SR_07	10	9.90	99.00%	19.67%	Pass
SR_08	5	NEG	–	–	Fail

a, Geometric mean of three determinations.

### Evaluation of clinical trial samples

**Figure 5:**
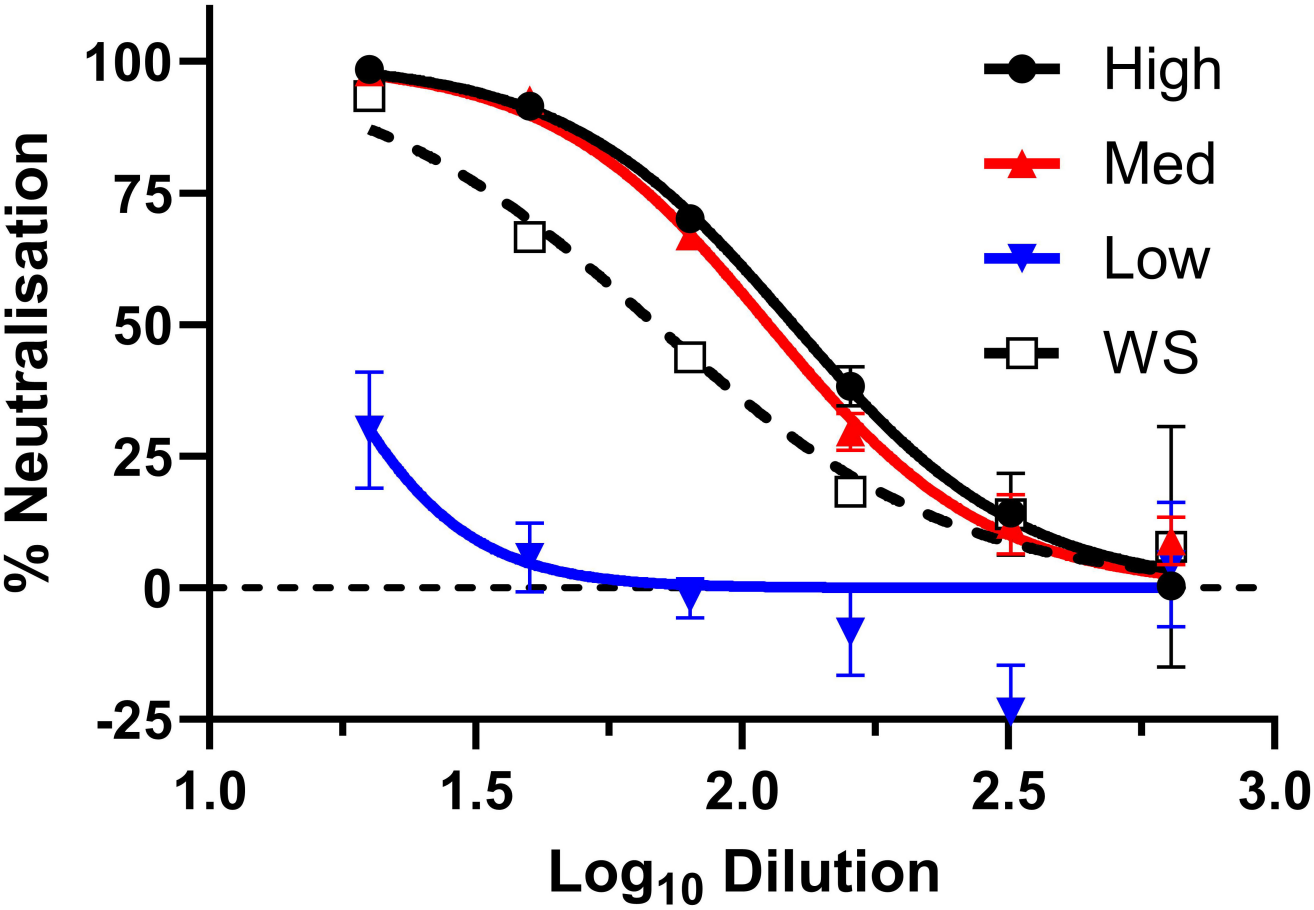
Neutralization curves of clinical trial samples. Three sample pools from a clinical trial were tested according to the established method. Symbols indicate the mean and standard deviation of technical duplicates. Lines represent the fitted four-parameter logistic curve. Data represent a composite of different plates from the same run. WS, representative working standard neutralization curve.

Three sample pools from a candidate Lassa vaccine clinical trial were tested using the established PVNA. The high- and medium-titer samples fell within the established range of the method (57.76 and 42.74 IU/mL, respectively). The low-titer sample fell below the LOD, though low-level neutralization was evident from the neutralization curve (**Figure 5**). Together, these results indicate that the established range of the method is appropriate for testing of clinical trial samples, though testing of a greater number of samples will be required to corroborate this result.

### Comparison of results between PVNA and authentic virus neutralization assay

**Figure 6:**
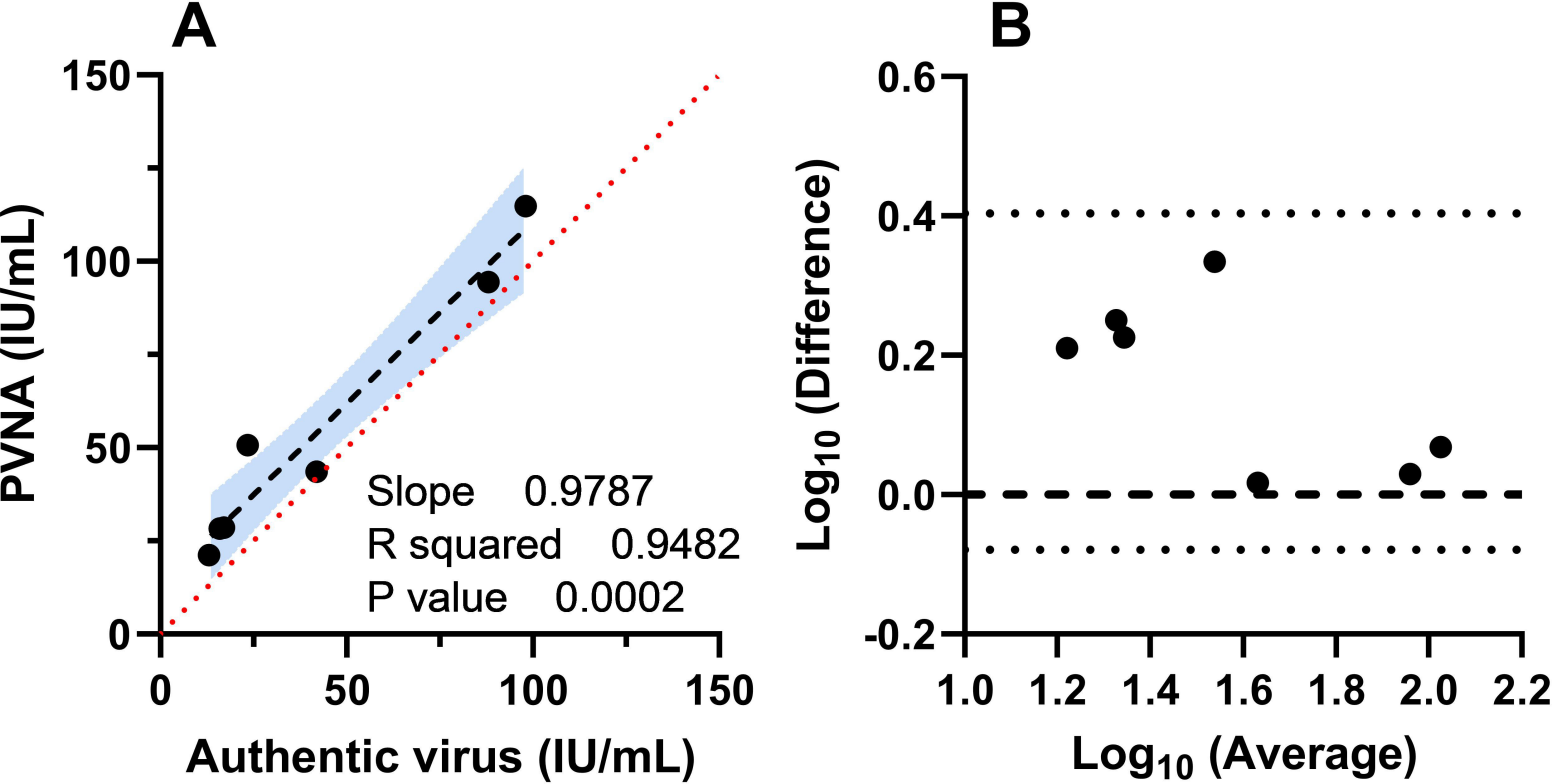
Comparison of PVNA and authentic virus neutralization assays. **(A)** Neutralizing antibody titers obtained in PVNA compared to those by the authentic virus neutralization method. The dashed line and blue shading indicate linear regression line and 95% confidence bands. The dotted red line indicates equivalence. **(B)** Bland–Altman analysis of results. The dashed line indicates zero point, i.e., perfect agreement; dotted lines indicate 95% limits of agreement.

The validated method was used to determine neutralization activity in a subset of samples previously tested in authentic virus neutralization assays during the collaborative study for the establishment of the IS 20/202 (18). Results obtained using the PVNA correlated well with authentic virus neutralization potencies (**Figure 6**, Pearson’s *r* = 0.974, *p* = 0.001, Spearman’s rho = 0.964, *p* < 0.001). Bland–Altman analysis indicated that the PVNA potencies were higher on average than those obtained by authentic virus neutralization, though the limits of agreement flanked zero.

### Robustness

A limited set of experiments was performed to estimate the impact of deliberated changes to conditions that may reasonably be expected to vary between laboratories. While individual sample potencies fluctuated, there was no trend for consistently higher or lower potencies under the conditions evaluated. Data are summarized in **Supplementary Figure 1**. Results of this robustness assessment were used to set tolerances in the Standard Operating Procedure method.

## Discussion

The development and evaluation of candidate vaccines in an outbreak setting can be greatly accelerated by the availability of validated, harmonized methods for quantification of key immune responses. We have developed and validated a PVNA for the quantification of anti-Lassa virus neutralizing antibodies. The method can be performed at CL-2 and requires approximately 3–4 h of operator time across a total duration of 28–30 h. Calibration of the WS against the WHO International Standard will allow for results generated using this method to be compared across different laboratories and over extended periods of time, facilitating harmonization of results and more informative comparison of different candidate vaccines. We encourage the use of the calibration approach described here to align with WHO guidelines and ensure optimal use of international standards. Ideally, sample potencies would also be determined by parallel line analysis against a WS. In practice, this approach is not always feasible, with the increased variability inherent in cell-based assays potentially impacting on parallelism. In this case, the use of a ratio approach, as described for the samples in this manuscript, is appropriate.

The difficulty in accessing large numbers of Lassa-positive samples limited our ability to fully characterize the limits of method sensitivity and specificity. While the estimates for sensitivity and specificity were both 100%, the lower confidence intervals were<80% due to the limited statistical power inherent in a small data set. Assuming 100% sensitivity and specificity, approximately 50 (each) positive and negative samples would be required to obtain confidence intervals with a lower limit of 90%. When additional samples become available, for example, through vaccine evaluation within the CEPI CLN, the sensitivity and specificity estimates described here can be refined.

The analytical range of the method was determined to be 10–100 IU/mL. A large majority of positive samples tested fell within this range. The samples available for the current study were mostly obtained from convalescent patients whereas the main intended use of the method is in the quantification of neutralizing antibody responses induced by immunization. Preliminary assessment of a limited number of samples from clinical trial participants indicated that the observed titers fell within the analytical range of the method. It will be important to establish the utility of the established range using a larger number of representative samples. In the event that a significant proportion of samples exceed the ULOQ of our method, a bridging exercise will be performed to validate an extended range. Alternatively, the linearity assessment can be extended to confirm that samples with titers higher than the ULOQ can be pre-diluted without compromising accuracy or precision.

The testing of a panel of samples within both the current study and the evaluation of the International Standard for Lassa antibodies (18) allowed us to compare our PVNA method with authentic virus neutralization assays. There was generally good agreement between the assays, with slightly higher values obtained by PVNA (**Figure 6**). A bias toward higher titers using PV systems has been observed for various viruses (7, 30, 31). Definitive mechanisms have not been identified but may include the fact that the readout from PV assays is obtained after a single cycle of infection while authentic virus assays are typically read following multiple rounds of viral replication. The latter likely requires a higher concentration of antibody to neutralize ongoing replication. Differences in glycoprotein density between the pseudotyped and authentic viruses may also affect the level of antibody required for neutralization. Further understanding and quantification of bias will be important in any assessment of correlates of protection based on neutralizing antibodies. It is emphasized that a limited number of samples (*n* = 7) were available for this study and analysis using an expanded panel would be required to confirm and extend the findings. Our results, nevertheless, indicate that PVNA potencies correlate well with authentic virus neutralization, and the incorporation of a WS calibrated against the International Standard in each assay format facilitates direct comparison of the methods.

A comprehensive robustness exercise was not undertaken due to limited sample and reagent availability. Preliminary assessment of key experimental conditions indicated that no consistent differences in sample potencies were observed when conditions were varied within ranges that might typically be expected due to inter-laboratory differences in operators, equipment, environmental conditions, etc. Method reproducibility is currently being assessed with a formal analytical method transfer. Furthermore, new batches of critical reagents, e.g., PV stocks, will be qualified by means of a formal bridging study to demonstrate fitness-for-purpose before use.

Multiple challenges exist in vaccine development in response to a pandemic. Logistical barriers and difficulties in obtaining and shipping samples from outbreak regions hinder progress, and the development and validation of new methods from scratch is time-consuming. We have addressed the latter issue by validating in advance a harmonized method for the evaluation of Lassa virus neutralizing responses. In the event of an outbreak of a novel viral pathogen, the method and validation approach described here can be rapidly adapted. We are currently in the process of transferring the established method to two additional partners within the CEPI CLN. This will increase capacity and resilience for testing of samples from clinical trials of Lassa vaccines. The inclusion of a WS calibrated against the WHO International Standard enables results to be expressed in IU/mL, enabling simple comparison of results obtained at different testing locations and, crucially, comparison of the results of different clinical trials. Increased comparability between studies facilitates informed decisions and supports regulatory approval; harmonization also contributes to the identification of correlates/surrogates of protection, which may inform patient management and public health guidelines.

## Data Availability

The original contributions presented in the study are included in the article/**Supplementary Material**. Further inquiries can be directed to the corresponding author.

## Supplementary material

Supplementary Figure 1 Assessment of method robustness. In each set of experiments, 2–4 samples were tested under the indicated conditions and potencies were calculated as described in the main text. **(A)** Various passages following resuscitation of cells. **(B)** Different cell seeding density prior to inoculation of neutralized virus. **(C)** Length of cell incubation at ambient temperature prior to transfer to a 37 °C incubator. **(D)** Time of virus neutralization. **(E)** Duration of infection. **(F)** Number of freeze–thaw cycles. Open circles and the dashed line indicate low positive control sample included in every plate.
