# Supplementary figures and images for: Development and validation of a pseudotyped virus neutralization assay for quantification of anti-Lassa virus neutralizing antibodies
Source: Front Immunol. 2026 Jun 16;17:1783813. doi: 10.3389/fimmu.2026.1783813 (PMC13314491; doi:10.3389/fimmu.2026.1783813)

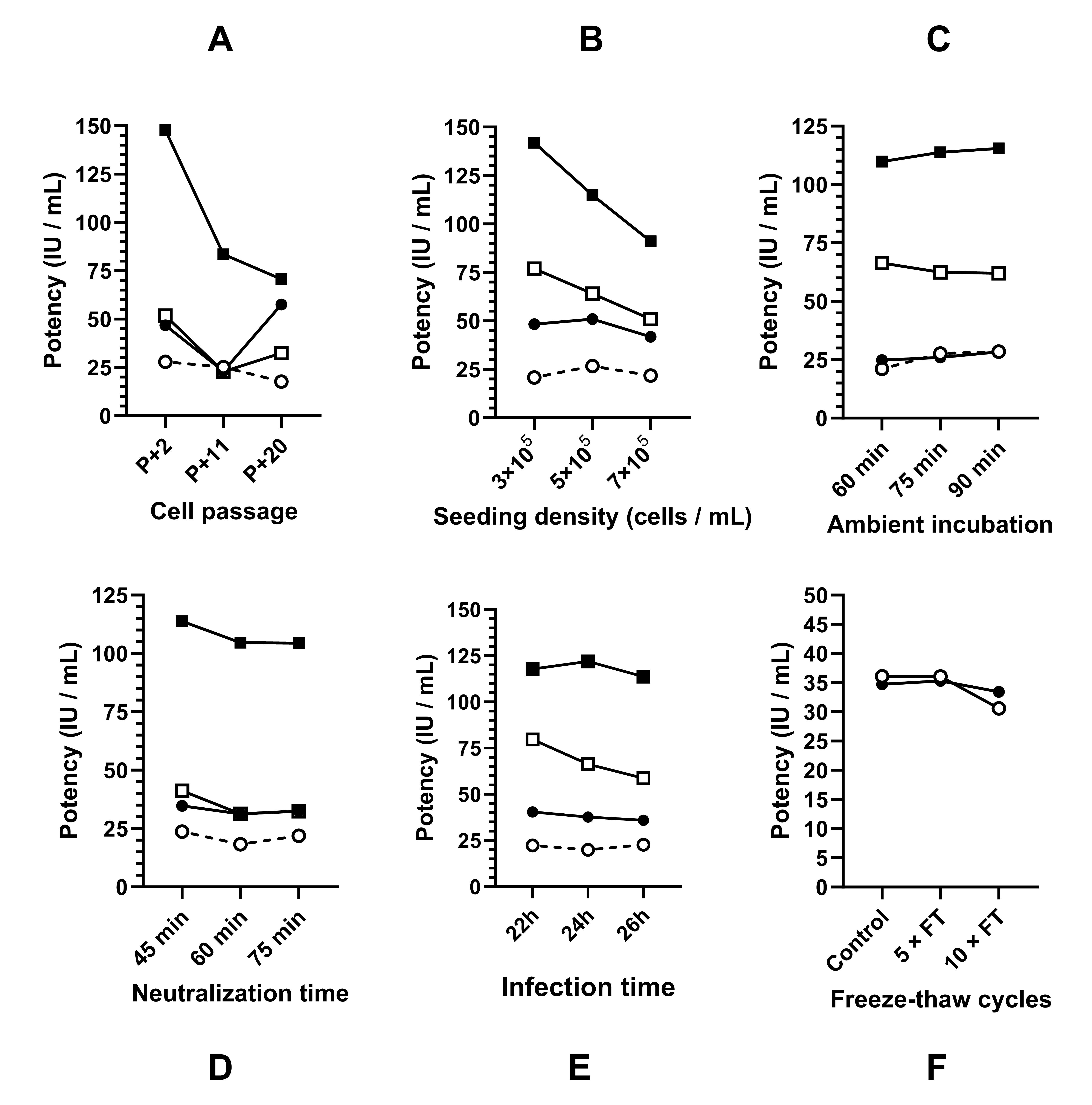

Supplement: Supplementary Figure 1 — Assessment of method robustness. In each set of experiments, 2–4 samples were tested under the indicated conditions and potencies were calculated as described in the main text. (A) Various passages following resuscitation of cells. (B) Different cell seeding density prior to inoculation of neutralized virus. (C) Length of cell incubation at ambient temperature prior to transfer to a 37 °C incubator. (D) Time of virus neutralization. (E) Duration of infection. (F) Number of freeze–thaw cycles. Open circles and the dashed line indicate low positive control sample included in every plate. [file Image1.jpeg]
